# Surgical Dressings*Synopsis of a lecture delivered before the Louisville Medical Society, June 18, 1885.

**Published:** 1885-08-20

**Authors:** Edward Von Donhoff

**Affiliations:** Louisville, Ky.


### SURGICAL DRESSINGS.*

*A Plea for the Early Abandonment of Dressings in the Treatment of Fractures of the Extremities and their Joints.*

BY EDWARD VON DONHOFF, A.M., M.D., LOUISVILLE, KY.

It is my intention to briefly present a matter in illustration of which I have had something to say on several former occasions, when cases that were accessible for purposes of public demonstration occurred in my practice. So far as I have had facility for looking up the literature of the subject, it appears that, either

*Synopsis of a lecture delivered before the Louisville Medical Society, June 18, 1885.

having tried (?) the method of which I propose to speak, "authors" have been disappointed by the results, or not having hit upon it at all in their practical or theoretical speculations, have had nothing to say of it. It seems somewhat remarkable, too, that surgical teachers, especially those who have personally investigated nature's *modus operandi* of the repair of fractured bones, should not, long since, have adapted their treatment of these injuries to her laws, which are so well calculated, when but slightly and wisely directed, to expedite and perfect cures in a way to fill at least the inexperienced in this matter with amazement. By the inexperienced I do not mean the tyro, nor do I use the expression in any way suggesting an absence of knowledge of current surgical laws of practice; but rather refer to those who, upon the mere presentation (?) of a single or even several exemplars of success, still hesitate to follow the lesson imparted, though the foundation for such a following be as demonstrable as a simple proposition in addition. I could wish, for the sake of the general benefit which it is intended to accomplish, that some one else, high in authority had inclined to speak long ago of the better, nay, brilliantly better results to be secured by the "early abandonment of dressings in the treatment of fractures," as compared with those obtained by the methods yet taught *ex cathedra*.

In one of the most recent editions of English surgery (that of Erichsen), under the head of "Fractures," occurs a concise but perfectly complete, for practical deductions, description of the process of repair after fracture of bones. Following this paragraph is the usual formula for treatment, and appended to this a vast array of splints, etc., and their complement of implied warning against premature (?) interference with what, in my humble estimation, constitutes the true bane of the subjects of this class of injuries. I refer to the overlong maintenance of "fixation," and the resultant temporary or permanent evil wrought thereby. As one looks over the field it would seem as if the very common occurrence of fractures had, contrary to what might fairly be expected, contributed but little, as evidenced by generally indorsed rules of practice, to a proper relationship of advancing practicable knowledge and methods of treatment. It is true that surgeons no longer "bleed" to facilitate reduction of a dislocation, or give sixty-grain doses of calomel, and other vaunted antiphlogistic medication, as a part of the approved treatment of fractures, but it *is* true also that limbs are confined in apparatus until the joints above and below the seat of fracture are stiffened and useless, and until the muscles are atrophied. It is also true that it is almost if not quite the expected thing that a joint, after facture involving it, should become anchylosed, or at best be permanently and seriously modified in its usefulness.

It is but fair to "surgery" and *surgeons* to add that much of this character of lamentable error is found outside their proper rank, but enough—a great deal—issues from hospitals and high places among general practitioners and surgeons to set the investigator agog. The deepest sense of active pity for the sufferers is the proper fruit of scientific inquiry into the causes; and this leads, in this instance, to a proper selection of means to the end desired. A more general application of these must avoid many lifetimes of travail for humanity and chagrin to practitioners.

The healing of a fractured human bone is, under ordinary circumstances, practically completed in five weeks ; and perfectly so in as many months. But the fixation of the fragments is accomplished much earlier—ten to fourteen days—and this fact is quite sufficient to constitute a safe stepping stone for the departure from former methods of treatment, which will be here advocated, based, as it is, upon a gratifying experience. Ten or fourteen days suffice for the perfect establishment of a firm ensheathing callous ; in other words, for the formation of a secure protection for the furtherance of subsequent natural processes of repair. It follows then that *mechanical* appurtenances are no longer essential, except as guards against violence, and, as will be shown, are positively harmful to the later management of the case, if unduly brought into requisition. In young adults the first four or six days are devoted by nature to the readjustment (after fracture) of injuries to the soft parts, and to the development of plasma which consists, in part, of the residuum of the extravasated blood, in part of contiguous shreds of the soft parts, and of new adventitious cellular elements. The periosteum is no longer distinctly traceable in the immediate vicinage of the "break," but has melted into the pultaceous mass by which it is surrounded. No effort at "fixation" is yet apparent. During this first reparative stage fixation (quiet) is necessary to prevent pain, caused by continued wounding of soft parts by bony points, and a tendency to displacements by muscular contraction.

The second stage of repair is itself mechanically protective against further injury. It consists in the condensation (hardening) of the mass before described, and the formation of a distinct ensheathing capsule, which envelopes it—fusiform in shape—and is continuous with the true periosteum contiguous to the newly formed membrane (?). At the end of, variably, the eighth, ninth, tenth, or, at farthest, fourteenth day, this natural fusiform splint is sufficiently strong to permit quite brusque handling of the limb under observation.

An irresistible desire led me to examine conclusively for my purpose all cases of fracture, of the long bones, which have come under my treatment during the past ten years, with a view to determining the exemplification last stated above, and it has in turn induced me to base my treatment during the past six years upon the data thus derived. My success has been so uniformly gratifying to my patients and myself that I cannot but feel congratulated upon the temerity exercised in the previous studies. My list of cases embraces subjects as young as four months and as old as eighty years, the intermediate ages being fairly represented also.

### TREATMENT AND ILLUSTRATIVE CASES.

My treatment with fixed apparatus differs only from others in that I most prefer for the first dressing, if accessible, strips of proper width—not to exceed four inches, even for the thigh—

made of sole-leather and dipped in hot water. In addition *absorbent cotton* and the necessary rollers.

This dressing is made to include, for the purpose of "resting" rather than "fixing," neighboring joints in fractures of the extremities. The position of the limb as a whole is adapted to the complete relaxation of its muscles, and the bandage is put on snugly but not tightly. At the time of the first dressing a perfect adjustment of the fragments is attempted and secured if possible. The dressing is then permitted to remain undisturbed, unless unlooked for symptoms arise, until the sixth day. Now this is replaced by a single sheet of suitably measured and cut leather, into which the limb is, after the most careful examination, placed and confined during the ensuing six or eight days, at the end of which the casement is kept removed during the day time, and adjusted again at night to prevent possible accidents during the involuntary motions of the sleeper. After the eleventh day passive motion of all the joints of the extremity is practiced daily and the patient is desired to make voluntary efforts. [*Particular stress is laid upon this direction, as regards surgeon and patient, in case of fracture of any of the joints of the extremities.*] After an average period of thirty days from the date of injury, the patients are dismissed with useful and perfectly physiological limbs. Nor does there seem to be any diminution in size, denoting musclar atrophy. [There are special emergencies of a diathetic and traumatic (compound fractures) character in which the good sense of the surgeon must suggest modifications of the above described conductment of cases in general.]

The following list of cases is offered without especial comment for want of sufficient space. The plan of treatment observed in each corresponds with the spirit of the rule laid down in the foregoing text-matter. Simple fractures of the shaft I have deemed it unnecessary to mention, except in one instance which was properly a "re-fracture" after a bad form of union and anchylosis of wrist and below the elbow (fibrous) after the *ordinary* treatment.

### CASE I.

Fracture of the internal tuberosity of the left femur. Man, aged forty years. Dr. J. A. Octerlony's patient. Dismissed in four weeks.

### CASE II.

Fracture of the neck of the humerus. Lady, aged thirty-seven years. Dr. Henderson's patient. Dismissed in thirty days.

### CASE III.

Colles's fracture. Old lady, aged eighty-five years. Dr. Samuel Branis's patient. Dismissed in four weeks.

### CASE IV.

Colles's fracture. Old lady, aged sixty-four years. Dr. W. Talbot Owen's patient. Dismissed in four weeks.

### CASE V.†

Comminuted fracture of the left elbow. Boy, aged fifteen years. Dismissed in twenty-eight days. Dr. S. B. Mills's patient. Dismissed in twenty-four days.

### CASE VI.

Intracapsular fracture of the left hip-joint. Patient aged fifty years. Dismissed in five weeks. Dr. S. Manly's patient.

### CASE VII.†

Comminuted fracture of the right elbow and fracture of the shaft of the right humerus and ulna. Boy, aged nine years. Exhibited to the Medical Chirurgical Society on the twelfth day after injury, having at that time "motion" both voluntary and passive. Dismissed in twenty-eight days.

### CASE VIII.

Osteotomy femoris, for anchylosis of the left hip, following morbus coxarius. Shown to Medical-Chirurgical Society in fourteen days, when the patient, aged twelve years, could take a few steps and could swing the limb, resting the body on the sound one. Dismissed, walking, in thirty days.

### CASE IX.

Osteotomy (bow-legs) both bones of the legs. Child, aged three years. Shown to Medical-Chirurgical Society, and dismissed in three weeks.

### CASE X.

Fractured fore-arm. Infant in arms. Dismissed in three weeks. Shown to the College of Physicians and Surgeons, Louisville.

### CASE XI.

Re-fracture of the fore-arm in consequence of malposition incident to imperfect treatment. Child, aged eight years. Patient (formerly) of Dr. H. F. Dismissed in three weeks with a straight arm.—*Louisville Medical News*.

---

†These cases were dressed at an angle of 125°, a point midway between complete extension and flexion. Both recovered perfect usefulness of the joint injured. Each was able to complete extension and nearly complete flexion at the end of three weeks, voluntarily, not a sign of stiffness even occurring during or after treatment of the shoulder, wrist, or finger-joints. Throughout the time succeeding the tenth day it is my custom to encourage the patient to turn about a round object with the fingers of the injured arm,

